# Comparative effectiveness of different core muscle training regimens for chronic non-specific low back pain: a systematic review and meta-analysis

**DOI:** 10.3389/fmed.2026.1814834

**Published:** 2026-04-20

**Authors:** Yang Liu, Yueyong Yu, Fan Lu, Junxuan Liu

**Affiliations:** College of Physical Education and Sports, Beijing Normal University, Beijing, China

**Keywords:** breathing training, chronic non-specific low back pain, core exercise, core stability, pilates, sling exercise therapy

## Abstract

**Objective:**

To systematically evaluate and compare the relative effectiveness of different core muscle training modalities in alleviating pain and improving function in individuals with chronic non-specific low back pain (CNLBP).

**Methods:**

Chinese and English language databases were systematically searched for randomized controlled trials involving individuals with CNLBP. Intervention groups received core training either alone or combined with other therapies, while control groups received usual rehabilitation or other exercise interventions. Primary outcomes were pain intensity and disability scores. Risk of bias was assessed using the Cochrane RoB 2.0 tool. A random-effects meta-analysis was performed using RevMan 5.4 software, with subgroup analyses conducted to examine the moderating effects of intervention type and intervention duration. Forest plots and funnel plots were generated using MATLAB-R2024.

**Results:**

Fifteen randomized controlled trialswere included. Meta-analysis revealed that core training significantly improved pain (SMD = –0.56, 95% CI: –1.08 to –0.03) and function (SMD = –0.81, 95% CI: –1.38 to –0.25). subgroup analyses indicated that combined interventions significantly improved function (SMD = –0.96, *P* = 0.002), although the test for subgroup differences between combined and single-modality interventions was not statistically significant (*P* = 0.96), and intervention durations of ≥ 8 weeks were associated with more pronounced analgesic effects. The overall quality of evidence was rated as “low,” primarily due to risk of bias and high heterogeneity.

**Conclusion:**

Current direct comparative evidence suggests that augmenting core training with additional rehabilitative components may confer greater benefits for functional improvement, while extending the intervention duration beyond eight weeks may optimize pain relief. Clinical decisions regarding training modality selection should be individualized based on the patient’s primary treatment goal. Further high-quality research is warranted to strengthen the evidence base for comparisons between specific training modalities.

**Systematic review registration:**

[https://www.crd.york.ac.uk/PROSPERO/view/CRD420251031252], identifier [CRD420251031252].

## 1 Introduction

Chronic non-specific low back pain (CNLBP) is a prevalent musculoskeletal condition characterized primarily by pain and functional impairment in the lumbar region, without attributable specific structural pathology (1). Its high global prevalence and strong disabling potential not only result in prolonged physical and psychological distress and diminished quality of life for affected individuals but also impose substantial socioeconomic burdens (1, 2). Current mainstream international clinical guidelines recommend non-pharmacological interventions centered on exercise therapy as the first-line rehabilitation strategy for CNLBP (3, 4). Among these, core muscle training—aimed at enhancing lumbopelvic stability—has been extensively demonstrated to effectively alleviate pain and improve function, with underlying mechanisms involving enhanced local muscle strength, optimized motor control, and modulation of neuromuscular adaptations (5, 6).

As rehabilitation practice has evolved, core training has diversified into multiple specific modalities, including core stability training (5), sling exercise therapy (7), Pilates (8), and control-focused training emphasizing respiratory and trunk coordination (9). Numerous randomized controlled trials have demonstrated that each of these core training modalities is superior to passive controls (e.g., usual care, placebo, or no intervention) (10). However, this paradigm of simply validating “efficacy” presents a limitation for clinical decision-making: when multiple core training modalities are all proven effective, clinicians require evidence on “which modality is superior” and “whether combined interventions yield synergistic benefits.” Although some head-to-head randomized controlled trials directly comparing different core training modalities have been conducted, their findings remain inconsistent, and a systematic quantitative synthesis is lacking.

Consequently, a critical gap exists in the current evidence base: the absence of a quantitative comprehensive evaluation, grounded in direct comparative data, of the relative effectiveness of different core training modalities for CNLBP. Traditional indirect comparisons or narrative reviews are insufficient to provide high-level evidence for such clinical decisions. To address this gap, the present study aims to conduct a systematic review and meta-analysis, rigorously including randomized controlled trials that directly compare two or more distinct core muscle training modalities. The primary objective is to quantitatively evaluate, through pairwise meta-analysis, the relative differences among training modalities (including comparisons between single modalities and between single versus combined modalities) in reducing pain and improving function. The findings are expected to provide direct, high-quality evidence to inform individualized and precise rehabilitation strategies for CNLBP in clinical practice.

## 2 Materials and methods

### 2.1 Study registration and reporting guidelines

The protocol for this study was registered with the PROSPERO International Prospective Register of Systematic Reviews prior to the initiation of the systematic review (registration number: CRD420251031252). The entire research process was designed, conducted, and reported in strict accordance with the Preferred Reporting Items for Systematic Reviews and Meta-Analyses (PRISMA) 2020 guidelines (11).

### 2.2 Inclusion and exclusion criteria

The eligibility criteria for this study were established following the PICO (Population, Intervention, Comparison, Outcomes) framework (11). Only randomized controlled trials (RCTs) meeting the following criteria were included: 1. participants were adults (≥ 18 years of age) with a clinical diagnosis of chronic non-specific low back pain (CNLBP); 2. the study design involved a direct comparison between two different active core muscle training regimens (e.g., core stability training vs. sling exercise training; single-modality core training vs. core training combined with breathing exercises or Pilates); 3. the study reported at least one primary outcome measure, including pain intensity [e.g., Visual Analogue Scale (VAS), Numerical Rating Scale (NRS)] or lumbar disability score [e.g., Oswestry Disability Index (ODI), Roland-Morris Disability Questionnaire (RMDQ)].

The exclusion criteria were as follows: 1. the intervention or control group included mixed treatments where the core component was not exercise-based, such as surgery, injections, or manual therapy; 2. non-randomized controlled trial designs (e.g., reviews, case reports, animal studies, conference abstracts); 3. missing or incomplete data preventing extraction of effect sizes for meta-analysis; 4. duplicate publications or reports of different follow-up periods from the same trial.

#### 2.2.1 Comparative designs

The included trials encompassed three types of direct comparisons: (1) intra-disciplinary comparisons (different modalities within the same core training discipline, e.g., reformer Pilates vs. mat Pilates); (2) cross-disciplinary comparisons (different core training modalities, e.g., sling exercise therapy vs. core stability training); and 3) augmentation comparisons (single-modality core training vs. the same regimen combined with adjunctive interventions, e.g., core stability plus breathing training). This classification provides a framework for interpreting the heterogeneity of pooled estimates.

### 2.3 Search strategy-4

A systematic literature search was conducted on December 31, 2024, covering six Chinese and English language databases: PubMed, Embase, the Cochrane Library, Web of Science, China National Knowledge Infrastructure (CNKI), and Wanfang Data Knowledge Service Platform. The search strategy was designed to maximize sensitivity by combining free-text terms with controlled vocabulary where applicable. For databases supporting controlled vocabulary, such as PubMed/Medline, Embase, and the Cochrane Library, a combination of Medical Subject Headings (MeSH)/Emtree terms and corresponding free-text words in titles and abstracts was used. The search syntax for PubMed was adapted as follows:

(((“Low Back Pain”[Mesh]) OR “Chronic Pain”[Mesh]) AND “Non-specific”[Title/Abstract]) OR (“Chronic Non-specific Low Back Pain”[Title/Abstract] OR “CNLBP”[Title/Abstract] OR “Mechanical Low Back Pain”[Title/Abstract])) AND ((“Core Training”[Title/Abstract] OR “Core Stability Training”[Title/Abstract] OR “Core Exercise”[Title/Abstract] OR “Core Strengthening”[Title/Abstract] OR “Sling Exercise Therapy”[Title/Abstract] OR “Pilates”[Title/Abstract] OR “Breathing Training”[Title/Abstract])) AND ((“Randomized Controlled Trial”[Publication Type] OR “Randomized Controlled Trial”[Title/Abstract] OR “RCT”[Title/Abstract] OR “Clinical Trial”[Title/Abstract]))

Similar search strategies, adapted to the specific vocabulary and syntax of each database, were applied in Embase (using Emtree terms), the Cochrane Library (using MeSH and keyword searches), and the other databases. The search timeframe covered the period from each database’s inception up to December 31, 2024. No restrictions were imposed regarding language or publication status. The complete search strategies for all databases are available from the corresponding author upon reasonable request.

### 2.4 Study selection and data extraction

Literature screening and data extraction were performed independently by two reviewers. Initial screening was conducted based on titles and abstracts, while full-text review was undertaken during the secondary screening phase to determine final study inclusion. Data were extracted using a standardized form, encompassing the following domains: 1. Study characteristics: authors, publication year, country, sample size, method of random sequence generation, allocation concealment, and blinding implementation. 2. Participant characteristics: for adult patients with a clinical diagnosis of chronic non-specific low back pain, data extracted included mean age, sex distribution, symptom duration, and baseline pain and function scores. 3. Intervention parameters: detailed extraction of the specific core training protocols employed in the experimental and control groups, including training type, weekly frequency, total intervention duration, and whether combined with other rehabilitative components. 4. Outcome measures: primary extraction focused on post-intervention mean values and standard deviations for pain intensity (VAS, NRS, or QVAS) and lumbar disability (ODI, RMDQ, or their culturally adapted versions). Where studies reported multiple follow-up time points, priority was given to data collected closest to the end of the primary intervention period.

Following independent data extraction by the two reviewers, discrepancies were resolved through cross-checking. In cases of unresolved disagreement, consensus was reached through discussion or by consulting a third reviewer to adjudicate, thereby ensuring data accuracy and consistency. All extracted data underwent final verification prior to analysis.

### 2.5 Risk of bias assessment-5

**FIGURE 1:**
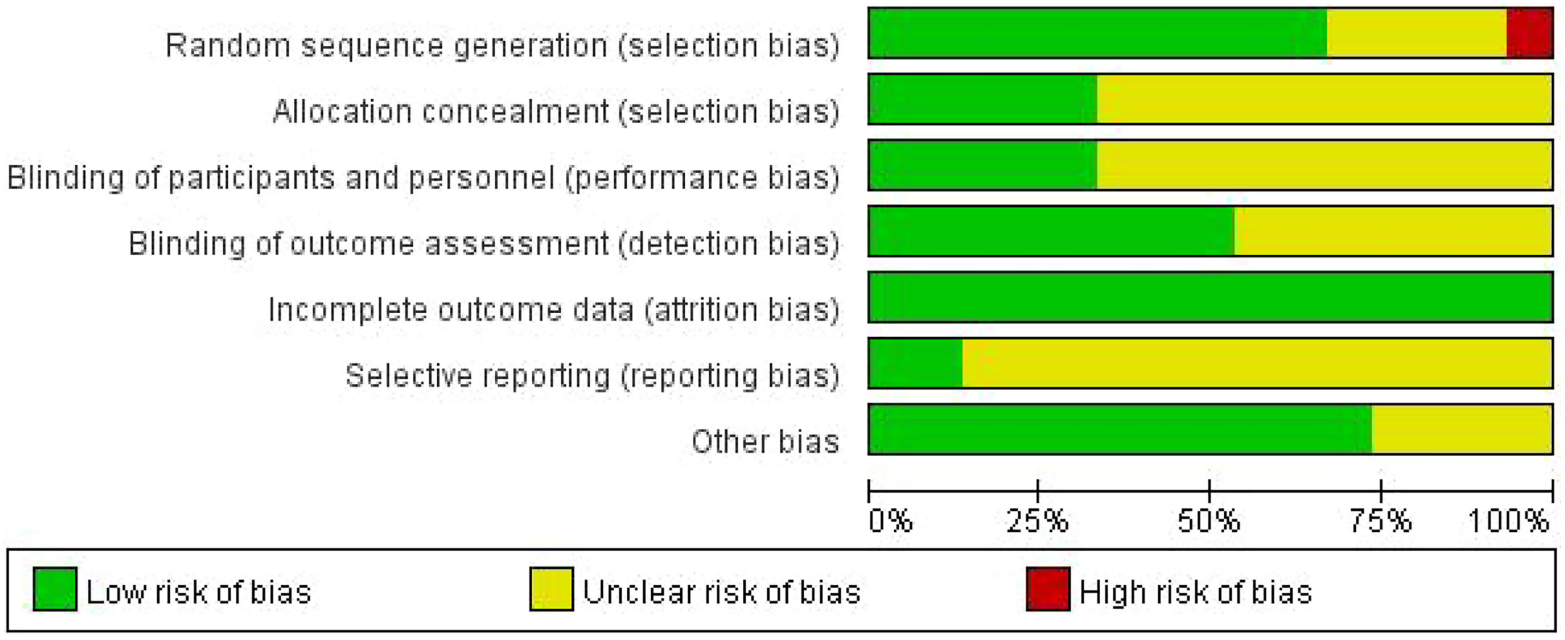
Risk assessment diagram for bias in the quality of 15 included studies.

**FIGURE 2:**
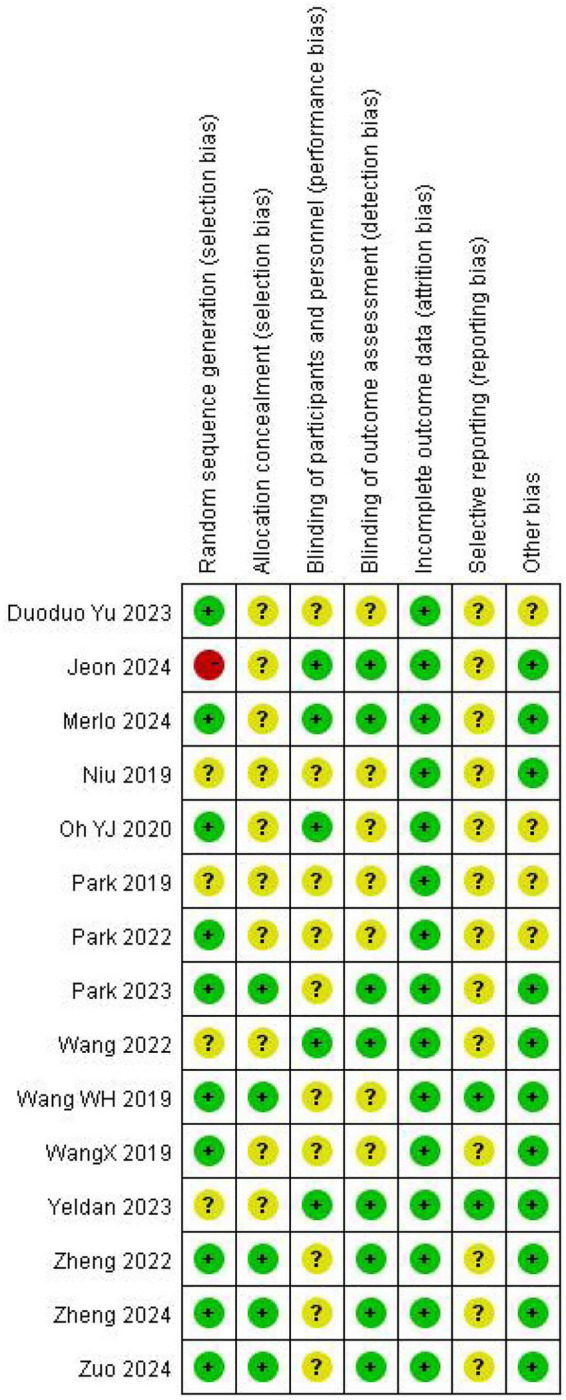
Summary chart of bias risk for the quality of 15 included studies.

The methodological quality of the included studies was evaluated using the Cochrane Collaboration’s Risk of Bias tool (RoB 1.0) as described in the Cochrane Handbook for Systematic Reviews of Interventions (Version 5.1.0) (Higgins, 2011) (10). Assessments were conducted independently by two reviewers, covering the following six domains: random sequence generation, allocation concealment, blinding of participants and personnel, blinding of outcome assessment, incomplete outcome data, selective reporting, and other sources of bias. Each domain was judged as presenting “low risk of bias,” “high risk of bias,” or “unclear risk of bias.” In cases of disagreement, consensus was reached through discussion or by consulting a third reviewer. The results of the assessment are presented in Figures 1, 2.

### 2.6 Data analysis

Statistical analyses were performed using Review Manager 5.4 software. For continuous outcomes (pain and function scores), the standardized mean difference (SMD) with its 95% confidence interval (95% CI) was used as the effect measure. Heterogeneity was assessed using the Cochrane Q test and the I^2^ statistic: a fixed-effects model was applied when *I*^2^ ≤ 50%, whereas a random-effects model (DerSimonian-Laird method) was employed when *I*^2^ > 50%. In the presence of substantial heterogeneity (*I*^2^ > 50%), pre-specified subgroup analyses were conducted to explore potential sources of heterogeneity.

To clarify the classification of interventions for subgroup analysis, an operational definition of “core training combined with other therapies” was established for this study: specifically, the integration of at least one non-core rehabilitative component explicitly and actively added to core muscle training (e.g., core stability training, sling exercise therapy, Pilates). These additional components may include breathing training (e.g., diaphragmatic breathing, respiratory resistance training), psychological or cognitive interventions (e.g., self-compassion training, cognitive behavioral strategies), physical agent modalities (e.g., interferential current therapy, photobiomodulation therapy), or other exercise modalities (e.g., traditional Chinese massage, progressive postural control exercises). In study designs where another therapy served as a baseline control (e.g., the control group receiving core training alone), and the experimental group received an additional intervention on the basis of the same core training protocol, the experimental group was classified as a combined intervention group.

Publication bias was assessed through visual inspection of funnel plots, supplemented by Egger’s test when the number of included studies was ≥ 10. Sensitivity analysis was performed using the leave-one-out method to examine the robustness of the pooled results. All analyses were conducted using final post-intervention measurements. In cases where standard deviations (SD) were not directly reported in the studies, they were calculated or estimated from standard errors, confidence intervals, or *P*-values in accordance with the Cochrane Handbook for Systematic Reviews of Interventions.

## 3 Results

### 3.1 Literature screening process

**FIGURE 3:**
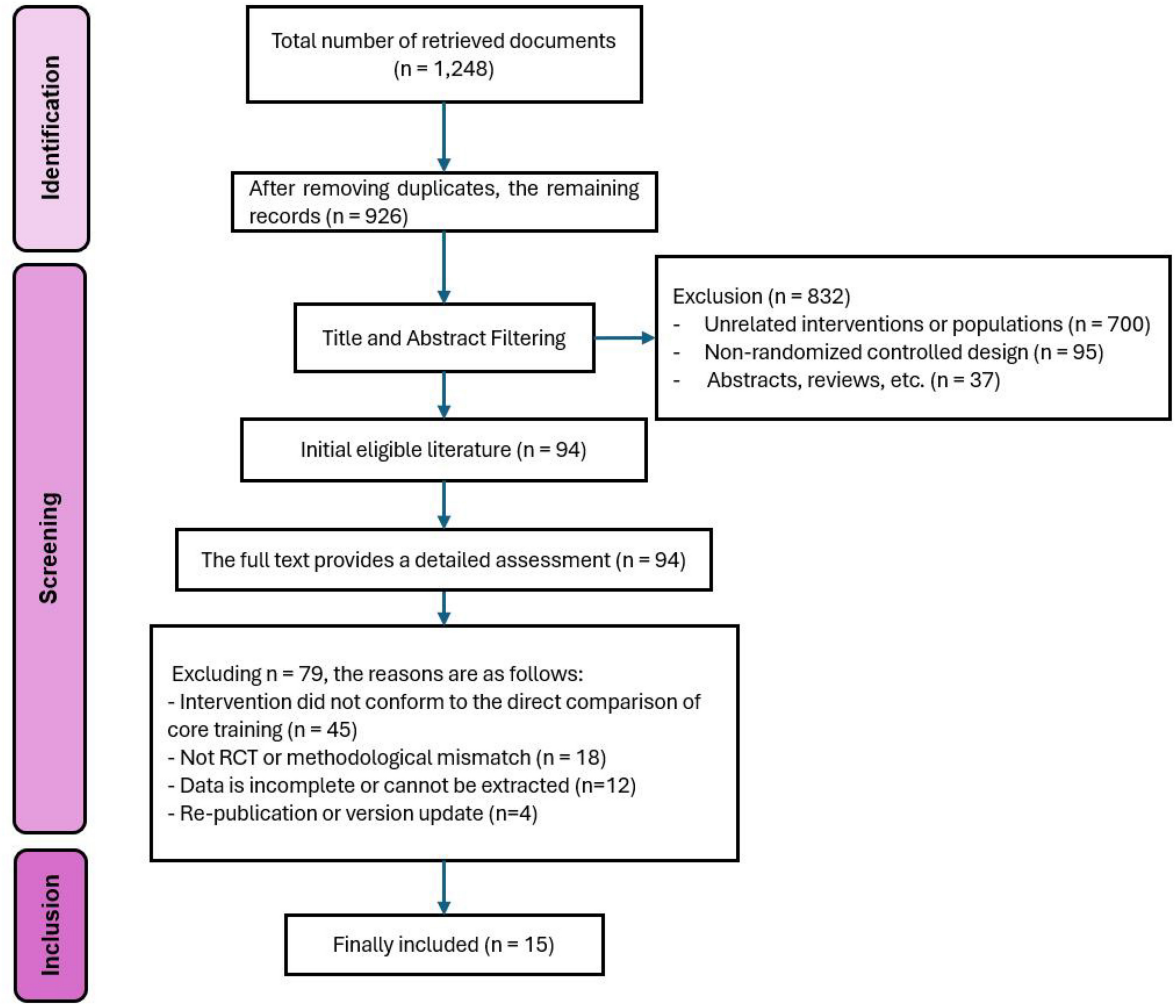
Flowchart of literature screening.

A systematic search of Chinese and English language databases yielded a total of 1,248 relevant records. Following deduplication, 322 duplicate records were excluded, leaving 926 records for initial screening. Through sequential review of titles and abstracts, 832 clearly irrelevant records were excluded, comprising primarily: 700 records where the research topic did not align with core training interventions or the chronic non-specific low back pain population; 95 records with non-randomized controlled trial designs; and 37 records classified as non-original research types, including conference abstracts, reviews, and commentaries. Subsequently, full texts of 94 studies that preliminarily met the inclusion criteria were retrieved and subjected to detailed evaluation, resulting in the exclusion of 79 studies that did not meet eligibility requirements. Reasons for exclusion included: interventions not involving direct comparisons of core training modalities (45 studies); study designs or methodological information not meeting randomized controlled trial requirements (18 studies); missing or unextractable key data (12 studies); and duplicate publications or different versions of the same trial (4 studies). Ultimately, 15 randomized controlled trials were included in this systematic review and meta-analysis. The detailed literature screening process is presented in Figure 3.

### 3.2 Table of basic characteristics of included studies

The basic characteristics of the 15 included randomized controlled trials are summarized in Table 1. For each study, detailed information is presented regarding study identification (authors, publication year, country), participant characteristics (sample size, age), intervention protocols (specific training modalities in experimental and control groups, intervention duration), and primary outcome measures, providing an essential foundation for subsequent analyses and interpretation of findings (Table 1).

**TABLE 1:** Basic information of included studies.

Included Study	Country	Sample Size	Age	Intervention Measures		Intervention Duration	Main outcome indicators
				Experimental Group	Control Group		
Zuo2024 (12)	China	53	28–56	CME + IFC	CME only; IFC only	12 weeks	VAS, ODI, SF-12
Wang 2022 (13)	China	34	18–40	PPCE	CSE	8 weeks	VAS, ODI, RMDQ
Yeldan 2023 (14)	Turkey	38	25–65	CSE + activity (biofeedback)	CSE (physiotherapist-assisted)	4 weeks	RODI, VAS
Zheng 2022 (15)	China	37	35.2 ± 11.1	mHealth-CSE + SCT	mHealth-CSE only	4 weeks	RMDQ, NRS
Park 2023 (16)	Korea	67	44.8 ± 10.8	SSE + ABB	SSE	24 weeks	VAS, ODI
Zheng 2024 (17)	China	62	35.3 ± 10.0	SCT + CSE	Cse	4 weeks	RMDQ, NRS
Duoduo Yu 2023 (18)	China	60	40.43 ± 8.42	CSE + BT	CSE	8 weeks	VAS, SCODI
Park 2019 (19)	Korea	43	18-65	PLSE + RRT	PLSE	4 weeks	NRS, K-ODI
Wang 2019 (20)	China	40	36.74 ± 2.68	SET	CSE	6 weeks	VAS, ODI
Niu 2019 (21)	China	42	30.82 ± 5.2	TCM + RML + SET	TCM + LMT	8 weeks	VAS, ODI
Wang X 2019 (22)	China	84	42.63 ± 9.60	SET + ACU	ACU	4 weeks	VAS, ODI
Jeon2024 (8)	Korea	30	35.92 ± 1.6	LSE (Pilates reformer)	LSE (mat)	8 weeks	VAS, ODI
Merlo 2024 (23)	Brazil	38	18-64	MP + PBMT	MP only	8 weeks	VAS, ODI, RMDQ
Oh YJ2020 (24)	Korea	60	40-49	ADIM-LSE + RRT	ADIM-LSE	4 weeks	QVAS, ODI-K
Park 2022 (25)	Korea	48	31.07 ± 6.82	SE + RRT	SE	5 weeks	QVAS, RMDQ

CME, Core Muscle Exercise; IFC, Interferential Current Therapy; PPCE, Progressive Postural Control Exercise; CSE, Core Stability Exercise; SCT, Self-Compassion Training; mHealth-CSE, Mobile Health-Based Core Stability Exercise; SSE, Spinal Stabilization Exercise; ABB, Abdominal Bracing; BT, Breathing Training; PLSE, Progressive Lumbar Stabilization Exercise; RRT, Respiratory Resistance Training; SET, Sling Exercise Training; TCM, Traditional Chinese Massage; RML, Rattan Moxibustion Liquid Liniment; LMT, Lumboabdominal Muscle Training; LSE, Lumbar Stabilization Exercise; MP, Mat Pilates; PBMT, Photobiomodulation Therapy; ADIM-LSE, Abdominal Draw-In Lumbar Stabilization Exercise; SE, Stabilization Exercise. K-ODI/ODI-K, Korean Version of Oswestry Disability Index;NRS, Numerical Rating Scale; ODI, Oswestry Disability Index, Oswestry; QVAS, Quadruple Visual Analog Scale; RMDQ, Roland-Morris Disability Questionnaire; RODI, Revised Oswestry Disability Index; SCODI - Simplified Chinese Version of Oswestry; Disability Index; SF-12, Short Form-12 Health Survey- SF-12; VAS, Visual Analog Scale; ACU, Acupuncture.

### 3.3 Assessment results of bias risk

The methodological quality assessment results for the 15 included randomized controlled trials are presented in Figures 1, 2. Overall, the included studies demonstrated acceptable quality in the reporting and implementation of certain key methodological aspects; however, notable deficiencies were observed in allocation concealment and blinding procedures. Specifically, regarding prevention of selection bias, the majority of studies (75%) were rated as low risk for random sequence generation, indicating generally reliable randomization procedures. Nevertheless, for the critical domain of allocation concealment, only 50% of studies were judged as low risk, with 25% each presenting unclear or high risk of bias. Concerning the minimization of performance and detection bias, blinding of participants and personnel (55% low risk) and blinding of outcome assessors (60% low risk) require improvement in both reporting and execution. With respect to attrition bias and reporting bias, management of incomplete outcome data (65% low risk) and selective reporting (70% low risk) was relatively better controlled. Additionally, the proportion of studies rated as low risk for “other bias” was the highest (80%).

### 3.4 Meta-analysis results

#### 3.4.1 Comparison of pain improvement effects among different core training modalities

**FIGURE 4:**
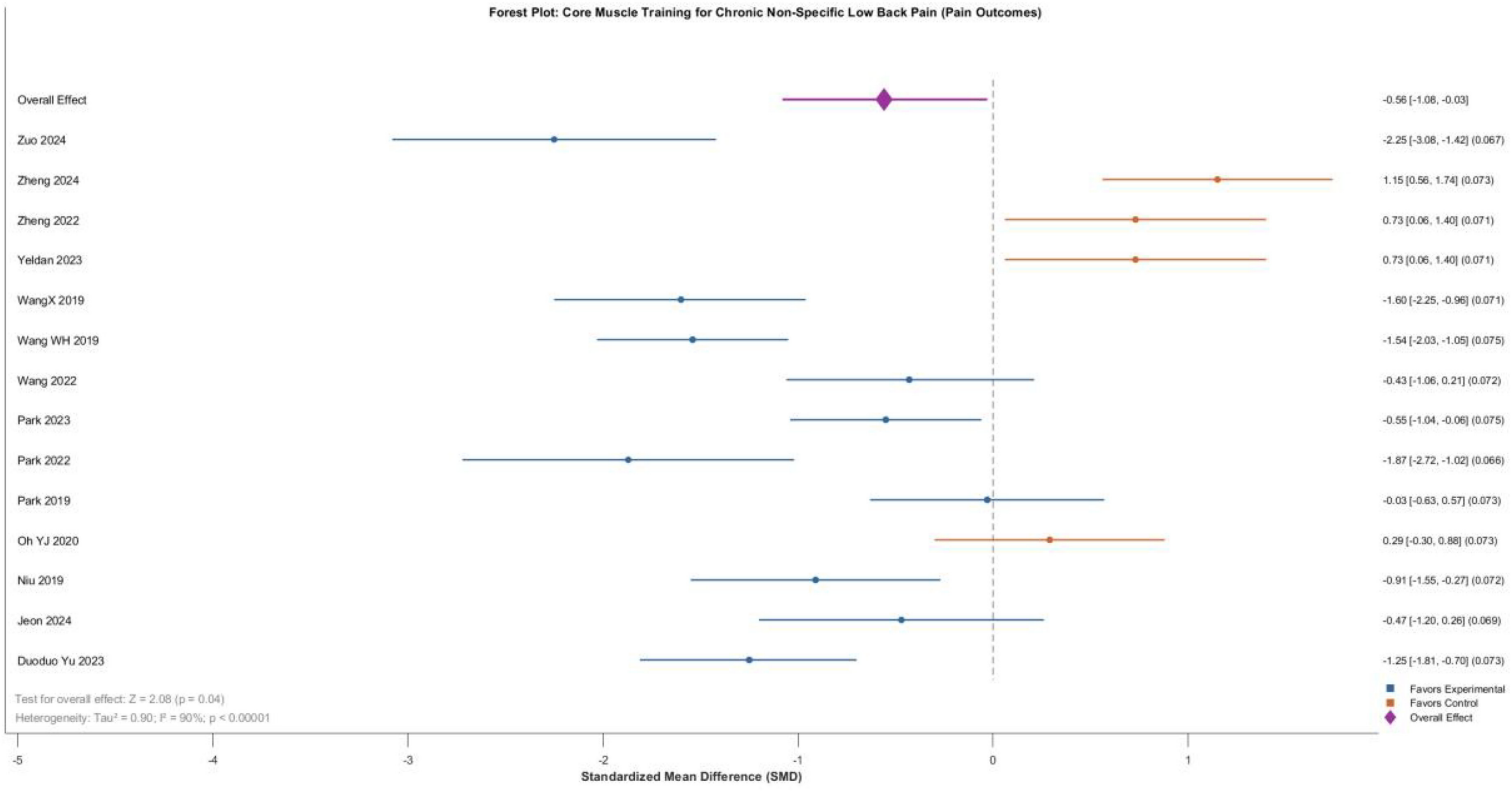
Forest plot comparing the pain improvement effects among different core training modes.

A total of 14 randomized controlled trials (excluding one study with non-estimable data) comprising 693 patients (343 in experimental groups, 350 in control groups) were included in this analysis. Meta-analysis using a random-effects model demonstrated that core training significantly improved pain symptoms in patients with chronic non-specific low back pain, with a standardized mean difference (SMD) of –0.56 (95% CI: –1.08 to –0.03), and the difference was statistically significant (*P* = 0.04). However, substantial heterogeneity was observed among the studies (*I*^2^ = 90%, *P* < 0.00001), indicating considerable variability in effect sizes across studies. This high heterogeneity suggests that the overall estimate represents an average across clinically diverse comparison types and should be interpreted with caution. Specifically, the majority of studies (11/14) favored core training for pain improvement, with several demonstrating large effect sizes, including Zuo et al. (12) (SMD = -2.25), Park et al. (25) (SMD = -1.87), and Wang et al. (20) (SMD = -1.54). In contrast, a minority of studies (3/14) showed superior pain improvement in the control group, although only the findings from Zheng et al. (17) reached statistical significance (Figure 4).

#### 3.4.2 Comparison of functional improvement effects among different core training modalities

**FIGURE 5:**
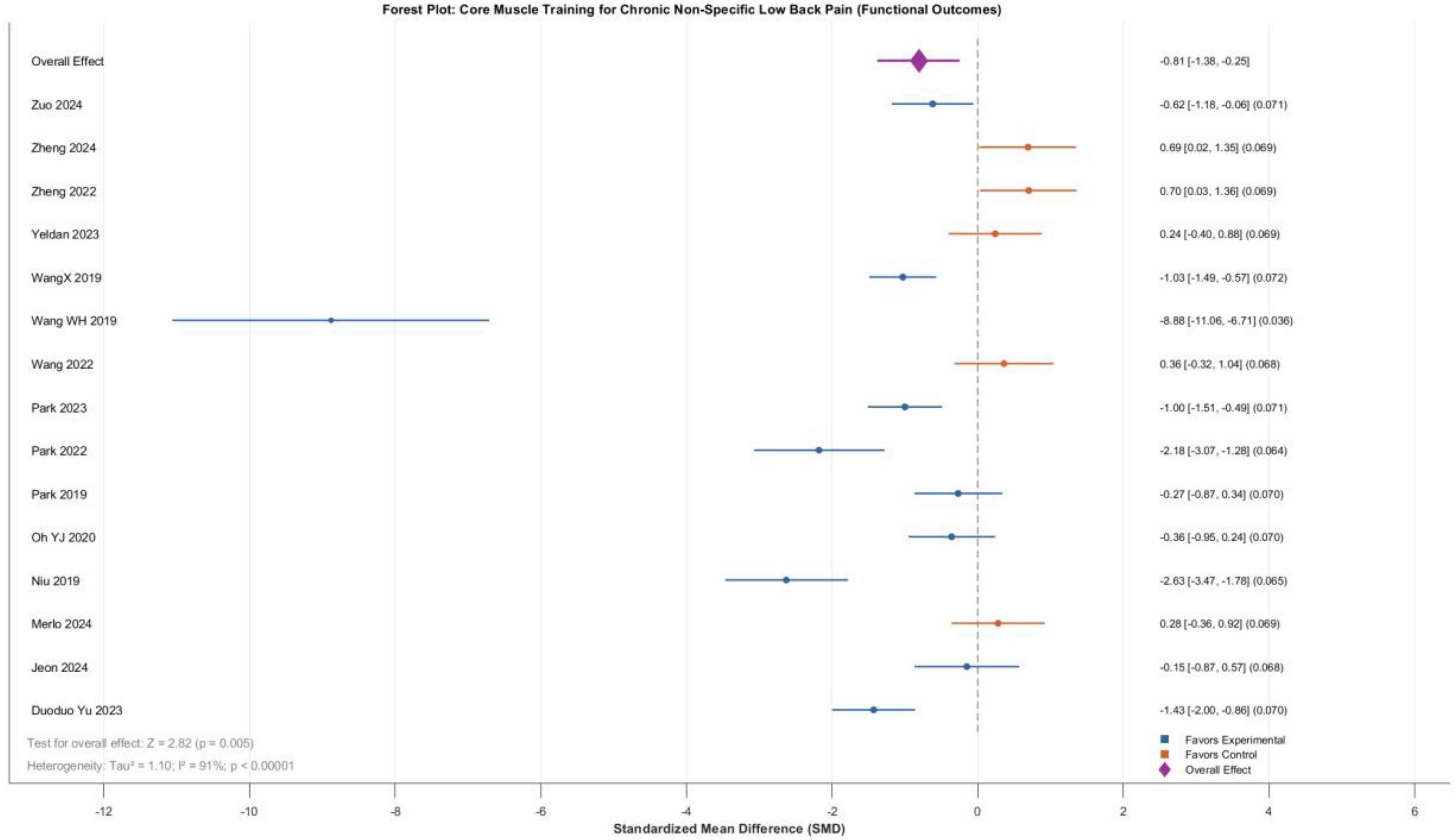
Forest plot comparing the functional improvement effects among different core training modes.

A total of 15 randomized controlled trials involving 677 patients with chronic non-specific low back pain (336 in experimental groups, 341 in control groups) were included in this analysis. Meta-analysis using a random-effects model revealed that core training yielded a standardized mean difference (SMD) of -0.81 (95% CI: -1.38 to -0.25) for functional improvement, with the overall effect test indicating statistical significance (*Z* = 2.82, *P* = 0.005), demonstrating that core training significantly improves functional status in patients with CNLBP. However, heterogeneity testing revealed substantial between-study heterogeneity (*I*^2^ = 91%, Tau^2^ = 1.10, Chi^2^ = 157.29, df = 14, *P* < 0.00001), reflecting considerable variability in effect sizes across studies. This high heterogeneity suggests that the overall estimate represents an average across clinically diverse comparison types and should be interpreted with caution. For instance, studies such as Wang et al. (20) demonstrated exceptionally large effect sizes (SMD = -8.88), albeit with relatively low statistical weight. Nevertheless, synthesis of the available evidence indicates that core training exerts significant beneficial effects on functional improvement in patients with chronic non-specific low back pain (Figure 5).

### 3.5 Subgroup analysis results

To explore potential moderators of intervention effects and account for the substantial heterogeneity observed (*I*^2^ > 90%), two prespecified subgroup analyses were conducted according to the study protocol. Studies were stratified by intervention complexity (single-modality core training vs. core training combined with other therapies) to determine whether integrating adjunctive components—such as breathing exercises, physical agents, or psychological strategies—enhances treatment outcomes, thereby informing optimal rehabilitation design. Studies were also stratified by intervention duration (< 8 weeks vs. ≥ 8 weeks) to examine whether longer treatment periods yield greater pain relief and functional improvement, providing empirical guidance for clinical dose prescription. All subgroup analyses employed random-effects models, with tests for subgroup differences used to assess statistical heterogeneity between subgroups.

#### 3.5.1 Subgroup analysis: single-modality core training versus combined interventions

##### 3.5.1.1 Pain improvement: single-modality versus combined interventions

**FIGURE 6:**
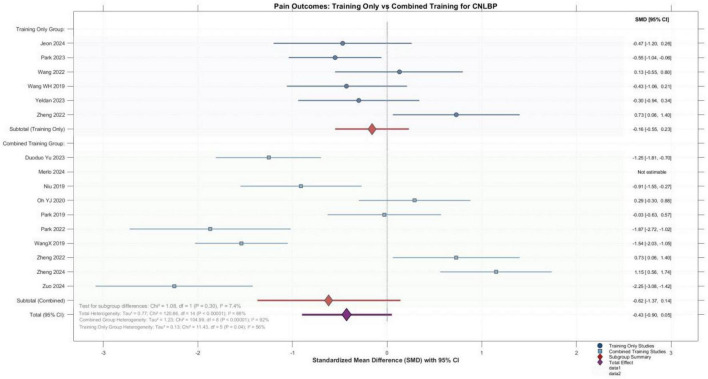
Forest plot showing the pain improvement effects of the single-modality group and the combined group.

Meta-analysis of 6 single-modality core training studies and 9 combined intervention studies (including one with non-estimable data) revealed an overall effect size of -0.43 (95% CI: -0.90 to 0.05) for pain improvement in patients with chronic non-specific low back pain, demonstrating marginal statistical significance (*P* = 0.08) and suggesting a trend toward pain reduction. To reduce clinical heterogeneity and provide more interpretable estimates, studies were grouped according to the nature of the intervention comparison. Subgroup analysis showed a pooled effect of -0.16 (95% CI: -0.55 to 0.23, *P* = 0.41) for single-modality training and -0.62 (95% CI: -1.37 to 0.14, *P* = 0.11) for combined interventions, with neither subgroup achieving statistical significance. The test for subgroup differences indicated no significant difference in effect sizes between the two intervention approaches (*P* = 0.30). Notably, substantial heterogeneity was observed within the combined intervention subgroup (*I*^2^ = 92%), reflecting considerable variability in the specific combination protocols employed and their corresponding effects across studies. Collectively, current evidence suggests that core training—whether delivered as a single modality or combined with other interventions—demonstrates a positive trend toward pain improvement; however, the effects have not yet reached consistently significant levels, and outcomes for combined interventions vary considerably across studies (Figure 6).

##### 3.5.1.2 Subgroup analysis of the functional improvement effects between the single-modality group and the combined group

**FIGURE 7:**
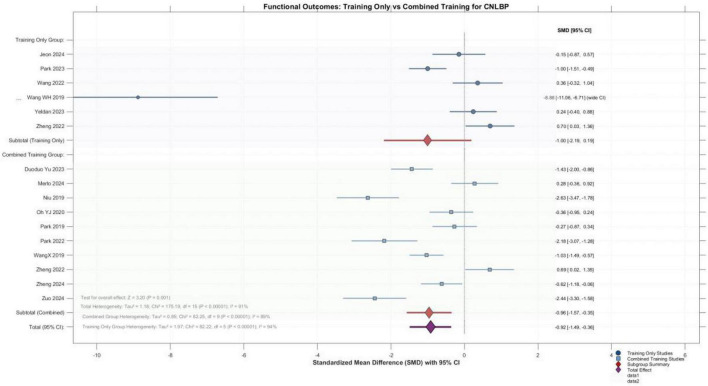
Forest plot of the pain improvement effects between the single-modality group and the combined group.

To reduce clinical heterogeneity and provide more interpretable estimates, studies were grouped according to the nature of the intervention comparison. Subgroup meta-analysis of single-modality core training (6 studies) and combined interventions (10 studies) revealed an overall effect size of -0.92 (95% CI: -1.49 to -0.36) for functional improvement in patients with chronic non-specific low back pain, with the overall effect test demonstrating high statistical significance (*Z* = 3.20, *P* = 0.001). Specifically, the combined interventions subgroup showed a pooled effect of -0.96 (95% CI: -1.57 to -0.35), indicating significant improvement (*Z* = 3.09, *P* = 0.002), whereas the single-modality subgroup yielded a pooled effect of -1.00 (95% CI: -2.19 to 0.19), which—despite demonstrating a large effect size—did not reach statistical significance (*Z* = 1.65, *P* = 0.10). The test for subgroup differences revealed no significant difference between the two intervention approaches (Chi^2^ = 0.00, *P* = 0.96). Notably, substantial heterogeneity was observed within both subgroups (single-modality *I*^2^ = 94%, combined *I*^2^ = 89%), with the extreme effect size from Wang et al. (20) (SMD = -8.88) exerting considerable influence on the pooled estimate for the single-modality group. Collectively, these findings indicate that combined core training significantly improves functional outcomes, whereas the effect of single-modality core training did not reach statistical significance in this analysis. However, the test for subgroup differences revealed no significant difference between the two intervention approaches (*P* = 0.96), indicating that the apparent advantage of combined interventions should be interpreted cautiously. Further high-quality research is warranted to confirm these findings (Figure 7).

#### 3.5.2 Subgroup analysis: intervention duration

##### 3.5.2.1 Pain improvement: short-term versus long-term interventions

**FIGURE 8:**
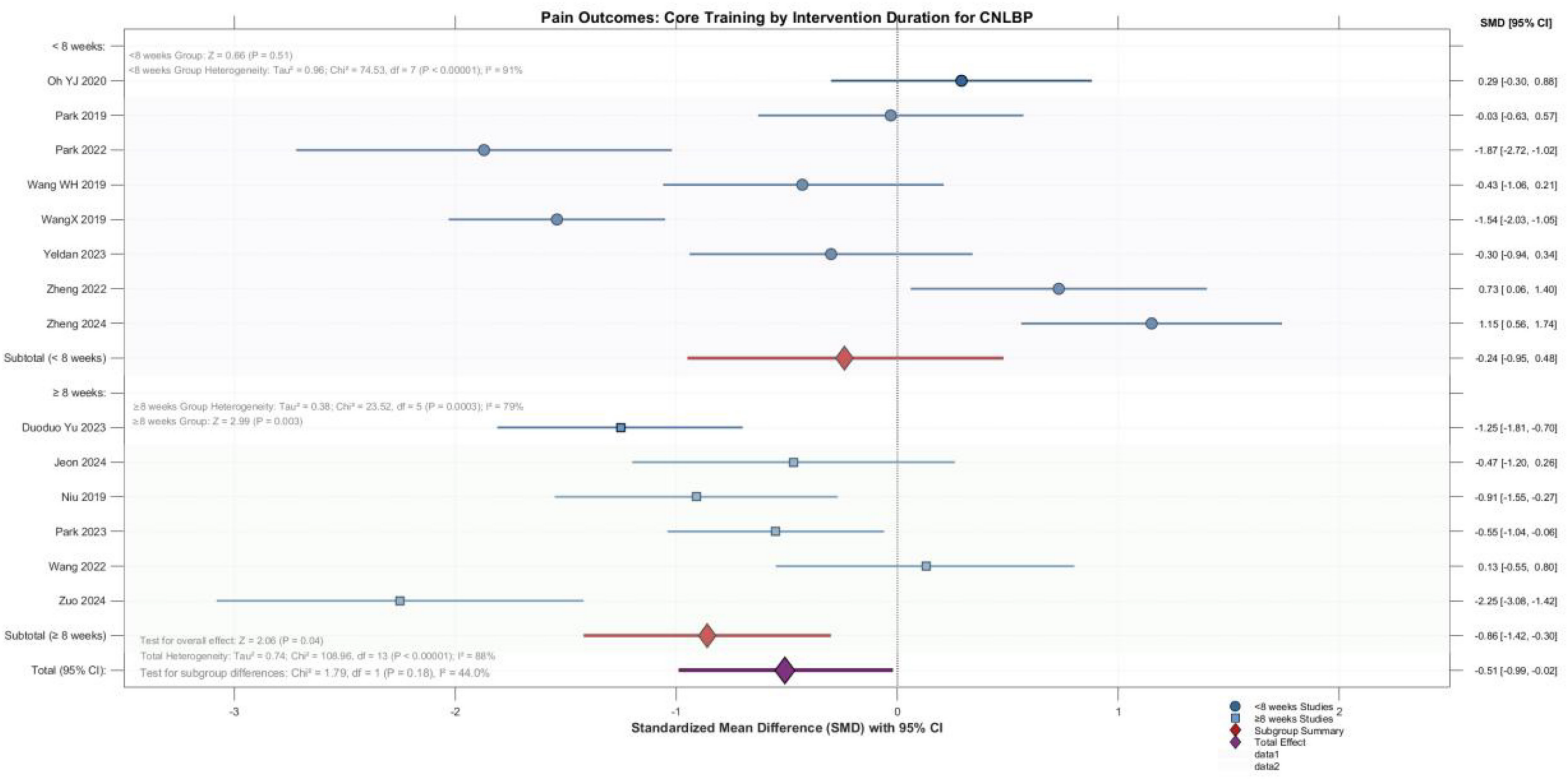
Forest plot of the effect of core muscle training during different intervention periods on pain reduction.

Subgroup analysis based on intervention duration included 14 studies. Overall meta-analysis demonstrated significant analgesic effects of core training, with a pooled effect size of -0.51 (95% CI: -0.99 to -0.02, *P* = 0.04). Subgroup analysis further revealed intervention duration as an important moderating factor: the ≥ 8 weeks subgroup exhibited clear and significant pain improvement, with an effect size of -0.86 (95% CI: -1.42 to -0.30, *P* = 0.003), whereas the < 8 weeks subgroup yielded an effect size of -0.24 (95% CI: -0.95 to 0.48), with confidence intervals crossing the null value and failing to reach statistical significance (*P* = 0.51). Although the test for subgroup differences did not reach statistical significance (*P* = 0.18), the point estimate for the ≥ 8 weeks subgroup was notably superior to that of the < 8 weeks subgroup and achieved statistical significance. Substantial heterogeneity persisted within both subgroups (*I*^2^ = 91 and 79%, respectively), suggesting that factors beyond intervention duration—such as training protocols and patient characteristics—may also influence treatment outcomes. Collectively, these findings suggest that extending core training duration to 8 weeks or longer may be important for achieving more stable and significant pain relief (Figure 8).

##### 3.5.2.2 Functional improvement: short-term versus long-term interventions

**FIGURE 9:**
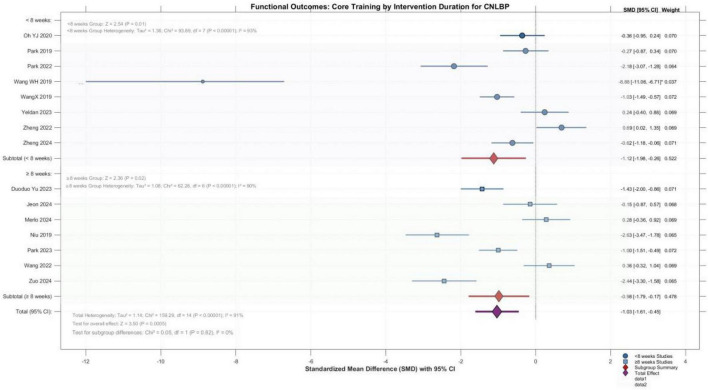
Forest plot of the effect of core muscle training during different intervention periods on pain reduction.

Subgroup analysis based on intervention duration for functional improvement included 15 studies, with one study excluded from this specific analysis due to unclear reporting of the intervention period, resulting in the inclusion of 14 studies. The analysis revealed an overall effect size of -1.03 (95% CI: -1.61 to -0.45), which was highly statistically significant (*Z* = 3.50, *P* = 0.0005). Subgroup analysis demonstrated that both the < 8 weeks subgroup (SMD = -1.12, 95% CI: -1.98 to -0.26, *P* = 0.01) and the ≥ 8 weeks subgroup (SMD = -0.98, 95% CI: -1.79 to -0.17, *P* = 0.02) exhibited significant functional improvement, with no statistically significant difference between the two subgroups (*P* = 0.82). Substantial heterogeneity persisted within both subgroups (*I*^2^ > 90%), suggesting that beyond intervention duration, other factors—including specific training protocols, frequency, intensity, and baseline patient characteristics—may also serve as important variables influencing treatment outcomes. Collectively, core muscle training demonstrates clear beneficial effects on functional improvement in patients with chronic non-specific low back pain, with these effects consistently observed across different intervention durations (Figure 9).

### 3.6 Sensitivity analysis results

Leave-one-out sensitivity analysis was conducted to assess the robustness of the pooled estimates for pain and functional improvement. Sequential exclusion of individual studies did not materially alter the direction of effects, with pooled SMDs consistently ranging from -0.48 to -0.65 and 95% confidence intervals remaining entirely below zero, confirming directional stability of the findings. However, point estimates showed some fluctuation, and substantial heterogeneity persisted (I^2^ consistently > 85%) across all iterations, indicating that studies with larger weights or divergent effect directions [e.g., (14, 17)] influenced the magnitude—but not the statistical significance—of the overall effect. These findings suggest that the primary meta-analysis results are relatively robust to the influence of individual studies, though the persistent high heterogeneity warrants cautious interpretation alongside subgroup analyses.

Notably, one study (20) exhibited an extremely large effect size for functional improvement (SMD = -8.88, 95% CI: -11.06 to -6.71) with a relatively low statistical weight (3.6%). A sensitivity analysis excluding this study was performed to assess its impact. After excluding Wang et al. (20), the pooled SMD for functional improvement changed from -0.81 (95% CI: -1.38 to -0.25, *I*^2^ = 91%) to -0.62 (95% CI: -1.05 to -0.19, *I*^2^ = 83%), remaining statistically significant (*P* = 0.005). Heterogeneity decreased from 91 to 83%, suggesting that this study contributed partially to the observed heterogeneity but did not solely drive the overall effect.

### 3.7 Evaluation of publication bias results

**FIGURE 10:**
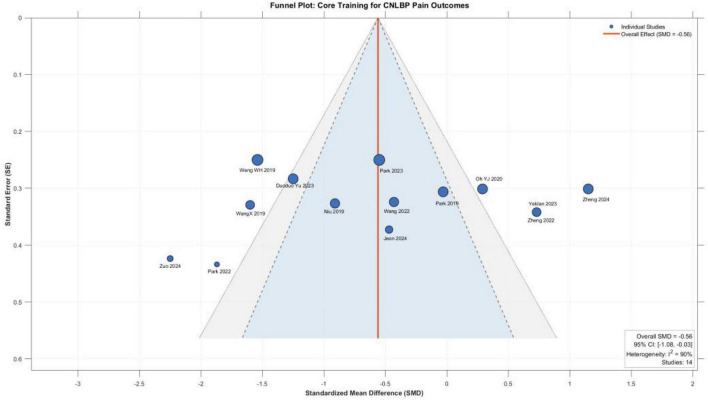
Funnel plot showing the effect of core muscle training on pain in patients with CNLBP.

**FIGURE 11:**
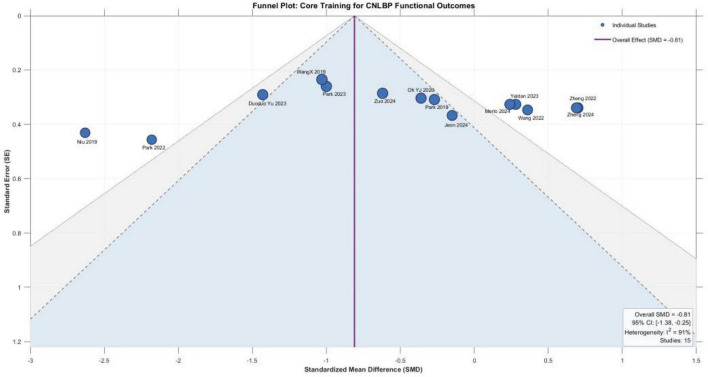
Funnel plot showing the functional impact of core muscle group training on patients CNLBP.

To assess potential publication bias, funnel plots were generated for the primary outcomes with ≥ 10 included studies (pain intensity and physical function), as presented in Figures 10, 11. Visual inspection revealed that the scatter points representing individual studies were approximately symmetrically distributed around the pooled effect sizes (pain SMD = -0.56, function SMD = -0.81) in both funnel plots, without exhibiting the typical asymmetry pattern characterized by marked clustering on one side or missing studies at the bottom.

In addition to visual inspection, Egger’s regression test was performed to quantitatively assess funnel plot asymmetry. For pain intensity (14 studies), Egger‘s test yielded an intercept of –1.84 (95% CI: –4.28 to 0.60, *t* = –1.64, *P* = 0.13), indicating no statistically significant evidence of publication bias. For functional improvement (15 studies), Egger‘s test showed an intercept of –2.31 (95% CI: –5.51 to 0.89, *t* = –1.56, *P* = 0.14), also suggesting no significant asymmetry.

It should be noted that the substantial heterogeneity observed in both meta-analyses (*I*^2^ > 90%) may influence the performance of Egger‘s test, as statistical tests for publication bias can be affected by between-study heterogeneity. Nevertheless, the combination of visual inspection and quantitative testing provides a comprehensive assessment of potential publication bias (Table 2).

**TABLE 2:** Egger’s test calculation result.

Outcome	Number of studies	Intercept	Standard error	95% CI	*t*-value	*P*-value
Pain Intensity	14	–1.84	1.12	–4.28 to 0.60	–1.64	0.13
Function score	15	–2.31	1.48	–5.51 to 0.89	–1.56	0.14

### 3.8 GRADE summary of findings

The quality of evidence was assessed using the GRADE approach. For the primary outcomes (pain intensity and physical function), the overall certainty of evidence was rated as low. This rating reflects downgrading by one level each for risk of bias (due to prevalent methodological limitations across studies, including inadequate reporting or implementation of random sequence generation, allocation concealment, and blinding procedures) and inconsistency (due to substantial statistical heterogeneity, with I^2^ exceeding 90%). Although no further downgrading was applied for indirectness, imprecision, or publication bias, the low certainty rating indicates that while core training may offer beneficial effects, confidence in the effect estimates is limited, and future high-quality research may alter these conclusions (Table 3).

**TABLE 3:** Summary of evidence and GRADE assessment.

Outcome measures	Number of included studies	Effect size (95% CI)	Risk of bias	Inconsistency	Indirectness	Imprecision	Bias of publication	Certainty of evidence	Importance
Pain intensity	15 (693)	SMD –0.56 (–1.08, –0.03)	Serious ^1^	Serious ^2^	Not serious	Not serious	Not serious^4^	low	Key point
Function score	15 (677)	SMD –0.81(–1.38, –0.25)	Serious ^1^	Serious ^2^	Not serious	Not serious^3^	Not serious ^4^	low	Key point

^1^ > 60% of the studies had blinding problems.

^2^ I^2^ > 50%.

^3^ Wide confidence intervals with important thresholds.

^4^ Visual inspection of funnel plots suggested approximate symmetry, consistent with Egger’s test results (*P* > 0.05).

## 4 Discussion

### 4.1 Interpretation of main findings

This study, based on direct comparative evidence, confirms that core muscle training exerts overall beneficial effects on pain and function in patients with chronic non-specific low back pain, with both analgesic effects (SMD = –0.56, 95% CI [–1.08, –0.03]) and functional improvements (SMD = –0.81, 95% CI [–1.38, –0.25]) reaching statistical significance. However, both outcomes were accompanied by substantial heterogeneity (*I*^2^ > 90%), indicating that treatment effects are moderated by multiple factors.

Further subgroup analyses provided more nuanced guidance for clinical decision-making. Regarding intervention strategies, both single-modality core training and combined interventions—integrating adjunctive components such as interferential current therapy (12), self-compassion training (15), breathing exercises (18, 24), or photobiomodulation therapy (23)—demonstrated trends toward improvement. Notably, combined interventions exhibited more stable and significant advantages in functional improvement (SMD = –0.96, *P* = 0.002), whereas the effect of single-modality training did not reach statistical significance (*P* = 0.10) (12, 15, 18, 23, 24). This suggests that integrating multimodal components—including neuromuscular, psychological-behavioral, or physical modalities—onto foundational strength and stability training may produce synergistic effects through multi-target mechanisms (e.g., modulating pain cognition, optimizing neuromuscular control), with particular potential for enhancing overall function and patient engagement.

Regarding intervention dosage, treatment duration emerged as a critical moderating variable for pain outcomes. Subgroup analysis demonstrated that protocols with intervention duration ≥ 8 weeks yielded clear and significant analgesic effects (SMD = –0.86, *P* = 0.003), whereas interventions lasting < 8 weeks failed to achieve statistical significance (*P* = 0.51) (12, 13, 18, 21). This finding aligns with the biological principles underlying exercise-induced neuromuscular adaptation and pain modulation, whereby sufficient cumulative stimulus and training duration constitute necessary conditions for reversing central sensitization and restructuring aberrant movement patterns. Notably, functional improvement remained significant across both duration subgroups (*P* < 0.05), suggesting that functional gains may exhibit a different dose-response relationship with intervention duration or may be more susceptible to non-temporal factors such as training specificity.

The pooled effect sizes represent an average across these distinct comparison types. While subgroup analyses explored some of this variability (e.g., augmentation vs. single-modality), the heterogeneity across comparison types warrants cautious interpretation of the overall estimates.

It must be emphasized that interpretation of these findings should consider the limitations revealed by GRADE evidence assessment. The overall certainty of evidence for primary outcomes was rated as “low,” primarily attributable to prevalent risk of bias (particularly in allocation concealment and blinding procedures) and substantial statistical heterogeneity. Therefore, although the current analysis supports the beneficial effects of core training—particularly combined and longer-duration protocols—confidence in the effect estimates remains limited. The observed heterogeneity profoundly reflects the diversity of patient characteristics and intervention details in clinical practice. Future research should focus on standardized reporting and identification of effect modifiers to advance the evidence base from establishing “average effectiveness” toward enabling “individualized applicability.” Recent network meta-analyses have demonstrated that various exercise modalities—including Pilates, sling exercises, motor control exercises, and core stabilization exercises—are effective in reducing pain and improving function in patients with CNLBP compared to conventional rehabilitation or no intervention (10).

A 2025 systematic review comparing core training modalities reported that all three approaches (Pilates, core stability training, and core resistance training) significantly improved pain relief (SMD = 0.70) compared to controls, with Pilates showing optimal effects for pain reduction and core resistance training demonstrating the most stable improvements in functional status (26). However, these analyses primarily compared active interventions against passive controls, leaving the question of relative effectiveness among different core training modalities incompletely addressed.

The current study extends this evidence base by focusing exclusively on head-to-head comparisons between active core training regimens. The findings confirm that core muscle training exerts overall beneficial effects on pain (SMD = –0.56) and function (SMD = –0.81) in patients with CNLBP. More importantly, subgroup analyses revealed that combined interventions (core training augmented with adjunctive therapies such as breathing exercises, psychological strategies, or physical agents) yielded more stable and significant improvements in functional outcomes (SMD = –0.96) compared to single-modality training. This finding aligns with the biopsychosocial model of CNLBP management, suggesting that multimodal approaches targeting both physical and psychological dimensions may confer synergistic benefits (27).

### 4.2 Mechanistic insights

The present findings offer preliminary insights into the potential mechanisms underlying core training for CNLBP. The observed advantage of combined interventions in improving function may be attributed to synergistic effects targeting multiple dimensions of the condition. While core stability training primarily aims to optimize lumbopelvic motor control, the addition of adjunctive components such as self-compassion training (15, 17) or breathing exercises (18, 24, 25)may concurrently address psychological factors (e.g., pain catastrophizing) or biomechanical factors (e.g., intra-abdominal pressure regulation). These multi-target effects could potentially enhance patient engagement and functional recovery more effectively than single-modality training alone.

The finding that interventions lasting ≥ 8 weeks yielded more pronounced analgesic effects aligns with the concept that exercise-induced adaptations may require sufficient cumulative stimulus. Short-term training may primarily induce peripheral changes, whereas longer-duration protocols are more likely to engage central mechanisms, including modulation of descending inhibitory pathways or reorganization of motor cortex representations. However, it is important to emphasize that these mechanistic interpretations remain speculative, as the included studies did not directly assess neurophysiological outcomes.

Furthermore, the substantial heterogeneity observed across studies (*I*^2^ > 90%) likely reflects the clinical reality that CNLBP is a multifactorial condition. Patients may present with varying degrees of central sensitization, neuromuscular dysfunction, or psychological distress, and may therefore respond differently to the same intervention. This heterogeneity underscores the need for future research to move beyond average treatment effects toward identifying which patient subgroups benefit most from specific core training modalities—a core principle of precision rehabilitation.

### 4.3 Study limitations

The findings and conclusions of this study should be interpreted within the context of several limitations inherent in the available evidence. Prevalent methodological shortcomings constituted a major source of bias risk. Inadequate reporting of allocation concealment and the general absence of blinding for participants and therapists may have compromised the objectivity of subjective outcome assessments, particularly for pain—a key factor contributing to the overall “low” certainty rating of the evidence (12–25). Furthermore, substantial clinical heterogeneity existed among the interventions themselves. Although collectively categorized as core training, the included protocols varied considerably in specific modalities, intensity, frequency, and whether they incorporated additional rehabilitative components, compounded by insufficient reporting standards. This diversity was a central factor driving the extreme statistical heterogeneity observed in the meta-analysis (*I*^2^ > 90%) and simultaneously poses challenges for translating the findings into specific clinical recommendations. Another notable concern is the temporal limitation of the evidence. The current body of research predominantly focuses on short-term intervention effects (4–8 weeks), with a lack of assessments regarding long-term efficacy maintenance or recurrence prevention, thereby constraining judgments on the enduring value of these interventions. Finally, it should be acknowledged that although the subgroup analyses conducted in this study were based on prespecified hypotheses, their results should be regarded as exploratory and hypothesis-generating rather than definitive conclusions. Future research necessitates more rigorously designed, head-to-head trials with standardized protocols and extended follow-up periods to validate the comparative effectiveness of different core training modalities and to further elucidate the individual conditions under which they exert optimal effects.

## 5 Conclusion

Synthesized direct comparative evidence indicates that core muscle training exerts beneficial effects on pain and function in patients with chronic non-specific low back pain. Among the various modalities, sling exercise training may offer greater potential for short-term pain relief. Combined interventions (core training augmented with adjunctive therapies) significantly improved functional outcomes, the difference compared to single-modality training did not reach statistical significance. To optimize therapeutic benefits, extending intervention duration to 8 weeks or longer is recommended. To optimize therapeutic benefits, extending intervention duration to 8 weeks or longer is recommended in clinical practice, with training modalities selected or combined individually based on the patient’s primary treatment goal—whether analgesia or functional restoration. This study provides direct comparative evidence to inform the clinical decision of “which core training modality is superior,” complementing existing evidence from network meta-analyses.” Future high-quality research is warranted to further validate the comparative effectiveness of specific modality pairings and to explore the individualized conditions under which they operate optimally.

## Data Availability

The original contributions presented in this study are included in the article/supplementary material, further inquiries can be directed to the corresponding author.
